# AXL is not essential for Zika virus infection in the mouse brain

**DOI:** 10.1038/emi.2017.10

**Published:** 2017-03-29

**Authors:** Feng Li, Pei-Rong Wang, Lin-Bing Qu, Chang-Hua Yi, Fu-Chun Zhang, Xiao-Ping Tang, Li-Guo Zhang, Ling Chen

**Affiliations:** 1 Institute of Infectious Diseases, Guangzhou Eighth People's Hospital, Guangzhou Medical University, Guangzhou 510060, Guangdong, China; 2 Key Laboratory of Infection and Immunity, Institute of Biophysics, Chinese Academy of Sciences, Beijing 100101, China; 3 Guangzhou Institutes of Biomedicine and Health, Chinese Academy of Sciences, Guangzhou 510530, Guangdong, China

**Dear Editor,**

Zika virus is a global threat to public health, particularly in pregnant women, as the virus can penetrate the placental barrier and infect embryos *in utero*, causing microcephaly and growth retardation in neonates.^1^ AXL receptor tyrosine kinase (AXL) was found to be a key for Zika virus infection in cultured 293T and A549 cells.^2, 3^ In addition, AXL is highly expressed by human radial glial cells, astrocytes, endothelial cells and microglia in the developing human cortex, and its expression in radial glia is conserved in the developing cortex of mice and ferrets, as well as in human stem cell-derived cerebral organoids.^4^ Therefore, AXL represents a promising candidate receptor and one of the most promising druggable targets against Zika virus. However, questions remain as to whether AXL is an essential receptor for Zika virus infection *in vivo*.

To answer this question, we generated BalB/c-*Axl*^−/−^ knockout mice using CRISPR/Cas9 technology (Figure 1A). Successful *Axl* knockout was further confirmed by fluorescence-activated cell sorting (Figure 1B). We then infected the BalB/c-*Axl*^−/−^ and BalB/c (WT) mice with a Zika GZ01 strain isolate (GenBank ID: KU761564.1). Neonatal pups (24–48 h after birth) were infected by intracerebral injection. The mice were killed at day 10 post-infection, and total RNA was extracted from the brains using the RNeasy lipid tissue mini kit (Qiagen, Cat No: 74804). Zika viral titers were measured with RT-PCR using the QuantiTect SYBR Green RT-PCR kit (Qiagen, Cat No: 204243) and Zika virus NS5 primers (GenBank ID: KX056898; NS5-F: 5′-TGG AGG CTG AGG AAG TTC TAG-3′ NS5-R: 5′-CTT CAC AAC GCA ATC ATC TCC ACT G-3′). Unexpectedly, not only was Zika virus detected in the brains of all *Axl*^−/−^ mice, but the Zika virus titers showed no significant difference (*P*>0.05) between BalB/c WT and BalB/c-*Axl*^−/−^ mice, reaching similar levels at doses of 1200, 120 and 1.2 PFU/mouse (Figure 1C). These results suggest that AXL deletion does not reduce Zika virus replication in mouse brains, indicating that AXL does not play an essential role—if any role at all—in Zika virus infection in mouse brains.

Our findings confirm that AXL is not the key receptor for Zika virus infection *in vivo* using an *Axl* knockout mouse model. Our results are corroborated by a recent study showing that genetic ablation of *Axl* does not protect human neural progenitor cells or cerebral organoids from Zika virus infection.^5^ This provides strong *in vivo* evidence for drug developers that AXL is not worth further investment as a target against Zika virus infection. Previous studies indicating that AXL might play a major role in Zika virus infection were conducted in cell culture,^2, 3^ suggesting that AXL might be necessary for infection in some special cell types. The role of AXL in infection with other viruses of the *Flaviviridae* family, such as the dengue, West Nile, yellow fever and Japanese encephalitis viruses, and the role of AXL in Zika-induced microcephaly need to be further investigated.

## Figures and Tables

**Figure 1:**
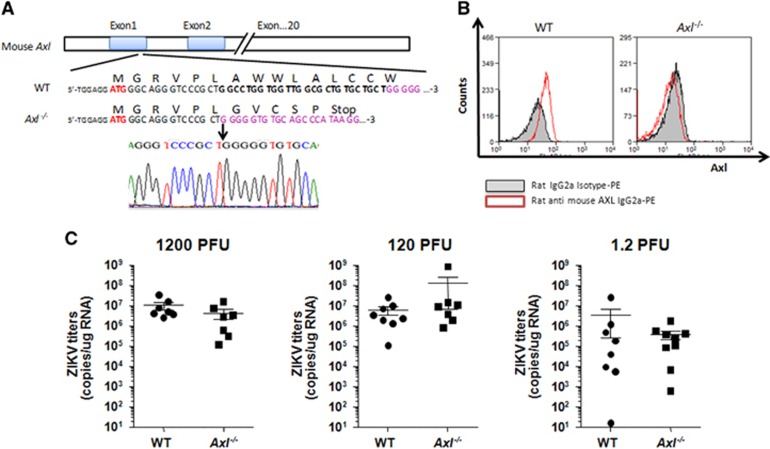
Zika virus infection in *Axl* knockout (*Axl*^−/−^) mice. (**A**) Generation of BalB/c-*Axl* knockout (*Axl*^−/−^) mice. A 26-bp deletion was introduced into exon 1 of *Axl* using CRISPR/Cas9, resulting in premature termination of translation (indicated). The deletion was confirmed by sequencing. (**B**) Confirmation of *Axl* knockout using fluorescence-activated cell sorting. Monocytes isolated from the spleens of WT and *Axl*^−/−^ mice were stained with rat anti-AXL antibody. (**C**) *Axl*^−/−^ mouse brains support Zika virus infection. Neonatal mice underwent intracerebral inoculation with Zika virus at 1200, 120 and 1.2 PFU/mouse. Zika virus titers in the brain were measured using real-time PCR. No significant difference (*P*>0.05) was observed between WT and *Axl*^−/−^ mice. plaque-forming unit, PFU; wild type, WT.
